# Glycation of H1 Histone by 3-Deoxyglucosone: Effects on Protein Structure and Generation of Different Advanced Glycation End Products

**DOI:** 10.1371/journal.pone.0130630

**Published:** 2015-06-29

**Authors:** Jalaluddin Mohammad Ashraf, Gulam Rabbani, Saheem Ahmad, Qambar Hasan, Rizwan Hasan Khan, Khursheed Alam, Inho Choi

**Affiliations:** 1 School of Biotechnology, Yeungnam University, Gyeongsan, Republic of Korea; 2 Interdisciplinary Biotechnology Unit, Aligarh Muslim University, Aligarh, India; 3 Department of Biotechnology, Integral University, Lucknow, India; 4 Department of Biochemistry, Jawaharlal Nehru Medical College, Aligarh Muslim University, Aligarh, India; University of South Florida College of Medicine, UNITED STATES

## Abstract

Advanced glycation end products (AGEs) culminate from the non-enzymatic reaction between a free carbonyl group of a reducing sugar and free amino group of proteins. 3-deoxyglucosone (3-DG) is one of the dicarbonyl species that rapidly forms several protein-AGE complexes that are believed to be involved in the pathogenesis of several diseases, particularly diabetic complications. In this study, the generation of AGEs (N^ε^-carboxymethyl lysine and pentosidine) by 3-DG in H1 histone protein was characterized by evaluating extent of side chain modification (lysine and arginine) and formation of Amadori products as well as carbonyl contents using several physicochemical techniques. Results strongly suggested that 3-DG is a potent glycating agent that forms various intermediates and AGEs during glycation reactions and affects the secondary structure of the H1 protein. Structural changes and AGE formation may influence the function of H1 histone and compromise chromatin structures in cases of secondary diabetic complications.

## Introduction

Advanced glycation end products (AGEs) are a heterogeneous group of complex molecules that include reactive derivatives generated during the non-enzymatic glycation process in which reducing sugars react with the free amino groups of amino acids, proteins, lipoproteins, and nucleic acids. The formation of a reversible Schiff base followed by the creation of covalently bound Amadori products along with a series of chemical reactions results in the irreversible formation of heterogeneous complex compounds known as AGEs [1]. Enhanced AGE development in cases of chronic hyperglycemia due to an increased rate of non-enzymatic glycation of macromolecules is known to cause tissue injuries and consequently aggravate secondary diabetic complications [2–4].

Previously, glucose was regarded as the major instigator of AGEs formation but several other sugar and non-sugar compounds have been found to be more reactive than glucose [5–8]. Among the non-sugar compounds, endogenous α-dicarbonyls are powerful glycating agents responsible for the generation of carbonyl stress, especially under diabetic conditions [9]. Indigenous α-dicarbonyl intermediates are formed through various mechanisms and pathways such as the glycolytic pathway, auto-oxidation of glucose, during all stages of Maillard reaction, and lipid peroxidation [10–13]. These entities are very reactive (20,000 times that of reducing sugars), and react swiftly with arginine and lysine residues on proteins; thus, AGEs can be formed even with extremely low concentrations of α-dicarbonyls [14, 15]. Among the α-dicarbonyls, 3-deoxyglucosone (3-DG), methylglyoxal (MG), and glyoxal are the major precursors of AGEs [16, 17]. 3-DG levels are elevated in patients with diabetes [18], and its role in the development of secondary diabetic complications has been reported [19].

Transcriptional activity of genes depends upon the packaging of chromatin by histone proteins [20]. Histones have been found to play important roles in the epigenetic regulation of gene expression [21, 22]. In particular, the linker histone (H1) is important for chromatin organization and the regulation of gene expression [23].

Several studies have demonstrated that histones are significantly vulnerable to nonenzymatic glycation. Gugliucci [24] provides evidence for the in vitro formation of both pentosidine and AGEs on histones. The same group revealed three folds higher AGE level in histones isolated from the liver of diabetic rats [25]. A similar result was also observed in diabetic patients [26]. In vitro analysis also showed glycation and glycoxidation of histone with ADP-ribose [27]. In the same way, mass spectrometry-based study of histones H1 and H2B has showed “glycoxidative” damage at different position and all of the identified glycated positions in both histones were lysine residues, [28, 29]. Recently, Talaz and co-workers [30] demonstrated the formation of AGEs in vitro glycation of H1 histones, core histones or total histones proteins with several sugars (glucose, ribose and fructose). Moreover, quite recent studies have shown the formation of AGEs by glycation of whole histones and H2A histone with methylglyoxal resulting in structural alteration in the proteins [31, 32]. Recently, we also produced evidence of AGE formation as well as structural change on histones in vitro [33, 34].

Because of the following factors, including permeability of nuclear membrane for lower weight molecules up to 5 kDa, peripheral position of histone H1 in chromatin, high content of lysine in H1 and long half life of this histone (4–5 month) make the H1 highly prone to be attacked by glycating agents. Being a small molecule, 3-DG can easily diffuse in nuclear membrane in turn can damage nuclear proteins by glycating it. To date, no extensive studies have been conducted to assess the glycation of H1 proteins by 3-DG. Therefore, the present study was conducted to examine the ability of 3-DG to glycate H1 histone protein. Generation of AGEs in H1 protein by 3-DG and secondary structural changes in the 3-DG-glycated H1 protein were evaluated by various physicochemical techniques.

## Materials and Methods

### Chemicals

3-DG, arginine, lysine, calf thymus H1 histone protein, sodium azide; 9,10-phenanthrenequinone, 2,4-dinitrophenyl hydrazine (DNPH); dialysis tubing, and other reagents/chemicals were obtained from Sigma Chemical Company (St. Louis, MO, USA). Nitroblue tetrazolium (NBT) was from Sisco Research Laboratories (India).

### Histone glycation by 3-DG

The glycation of calf thymus H1 histone (1 mg/mL) was carried out by incubation with 10 μM 3-DG and 10 mM sodium phosphate saline buffer (pH 7.4) at 37°C for 14 day. Native H1 histone was incubated under the same conditions but without 3-DG as a control. Dialysis was performed at 4°C against sodium phosphate saline buffer at pH 7.4 to remove unreacted 3-DG in the solution. The samples were stored at -80°C for later use.

### Assessment of free amino groups by a fluorescamine assay

Lysine side chain modifications were evaluated with a fluorescamine assay as previously described with slight modification [35–37]. Briefly, 5 μL (1 mg/mL) of a solution containing 3-DG-glycated H1 histone, 100 μL of 100 mM Na_2_HPO_4_, 45 μL aqua dest, and 50 μL fluorescamine reagent (1 mM fluorescamine in acetonitrile) were mixed and incubated for 10 min in the dark at room temperature. Fluorescence of the sample was measured at excitation/emission wavelengths of 390/490 nm on FLUORO-STAR plate reader (BMG, Germany). N-α-acetyl-lysine at concentrations of 0 to 1.5 mM was used as the standard to determine the lysine content of the protein solution. Fresh H1 protein (1 mg/mL) and PBS solution were used as controls for AGE-H1 histone and background fluorescence, respectively.

### Examination of free arginine side chains with a 9,10-phenanthrenequinone assay

Free arginine content was measured as previously described [38, 37]. Briefly, 50 μL of H1 protein (1 mg/mL) was mixed with 150 μL (150 μM) of 9,10-phenanthrenequinone reagent (dissolved in 100% ethanol) followed by the addition of 25 μL NaOH (2 N). The final reaction containing the proteins minus 9,10-phenanthrenequinone was used to correct the AGE fluorescence results. 3-DG-glycated H1 protein was incubated at 60°C for 3 h after which 40 μL of the reaction was transferred to a multiple-well plate and mixed with 40 μL of 1.2 N HCl. Fluorescence was measured at excitation/emission wavelengths of 312/395 nm after incubating the samples for 1 h in the dark at 25°C on FLUORO-STAR plate reader (BMG, Germany). N-α-acetyl-arginine (0 to 0.4 mM) was used as a standard to measure the arginine content. H1 histone (1 mg/mL) and PBS solution were used as controls for the AGE-H1 histone and background fluorescence, respectively.

### Detection of Amadori products in 3-DG-glycated H1 histone

NBT dye was used to detect Amadori product (fructosamine) in the 3-DG-glycated H1 protein as previously described with minor modification [39, 33]. Briefly, samples were mixed at a ratio of 1:10 with sodium carbonate-bicarbonate buffer (100 mM, pH 10.8) containing 0.25 mM NBT and then incubated at 37°C for 45 min. Absorbance was measured at 525 nm relative to distilled water. Amadori product concentration (nM/mL) was determined by multiplying the absorbance by a molar extinction coefficient of 12.64.

### Evaluation of carbonyl contents

Carbonyl contents of native and 3-DG-glycated H1 histone samples were determined as previously described with slight modifications [40, 33]. Briefly, 15 μM 3-DG-glycated and native H1 histones were dissolved in 10 mM 2,4-dinitrophenyl hydrazine (DNPH) solution in 2 N HCl. The samples were then vortexed for 1 h at room temperature, subjected to precipitation with 0.5 mL of 20% (v/v) TCA (Trichloroacetic acid), and centrifuged at 11,000 × g for 3 min at 4°C.

The resulting pellet was washed with 1 mL of an ethanol-ethyl acetic acid mixture (1:1; v:v) to remove extra DNPH reagent. Next, the samples were incubated at room temperature for 10 min, and then centrifuged at 11,000 × g for 5 min at 4°C. The pellet was washed twice with the ethanol-ethyl acetic acid mixture and suspended in 1 mL of 6 M guanidium hydrochloride dissolved in 20 mM potassium phosphate buffer (pH 2.3, adjusted with trifluoroacetic acid). The samples were subsequently incubated at 37°C for 15–30 min to dissolve the proteins. Carbonyl contents in the supernatant were measured relative to 6 M guanidium hydrochloride (as a blank) using a molar extinction coefficient of 22,000 M^-1^ cm^-1^ at 370 nm. Protein carbonyl contents were expressed as nmol/mg of protein.

### Evaluation of carboxymethyl lysine (CML) content by an ELISA

CML generation was measured with an ELISA as described previously with slight modification [41, 37] using 2,2’-azinobis (3-ethylbenzothiazoline-6-sulfonic acid) as the detection agent [S1 Fig]. A solution containing native H1 protein (1 mg/mL) and PBS were used as controls. An ELX800 multi-well plate reader (BioTek Instruments, USA) measured the absorbance of the samples at 405 nm.

### Determination of AGE-CML and pentosidine with a chromatographic assay

CML and pentosidine concentrations in the sample were estimated by high performance liquid chromotography (HPLC) as previously described [42, 33]. For enzymatic hydrolysis, all procedures were carried out under nitrogen. Briefly, 100 μg of samples were diluted with 20 μL of water followed by the addition of 25 μL (40 mM) HCl, 5 μL of a pepsin solution (2 mg/mL in 20 mM HCl), and 5 μL of a thymol solution (2 mg/mL in 20 mM HCl). The samples were incubated at 37°C for 24 h. The reactions were then neutralized with 25 μL of 0.5 M potassium phosphate buffer (pH 7.4) and 5 μL (260 mM) KOH. Next, 5 μL of pronase E (2 mg/mL in 10 mM KH_2_PO_4_, pH 7.4) were added and the reactions were incubated at 37°C for 24 h. Subsequently, 5 μL of an aminopeptidase solution (2 mg/mL in 10 mM KH_2_PO_4_, pH 7.4) and 5 μL of a prolidase solution (2 mg/mL in 10 mM KH_2_PO_4_, pH 7.4) were added, and the samples were incubated at 37°C for 48 h. Afterward, 50 μL enzymatic hydrolysate (equivalent to 50 μg of protein) were placed in 1-mL glass vials, and 10 μL internal standard (α-aminobutyric acid, 100 nmol/mL), 40 μL water, 100 μL aminoquinolyl-N-hydroxysuccimidyl-carbamate (AQC) derivatizing buffer (500 mM borate buffer and 400 μM DTPA, pH 8.8), and 200 μL AQC (6-Aminoquinolyl-N-hydroxysuccinimidylcarbamate) (10 mM in acetonitrile) were added. Calibration standards contained AGE standards (0–1000 pM) and amino acids (0–20 nM). The test and calibration standard samples were incubated at 55°C for 10 min after which the samples were lyophilized and reconstituted in 100 μL water. AQC-labelled hydrolysates were filtered by centrifugal filtration (0.2-μm pore) and the filtrates were then analyzed by reverse-phase HPLC. The Waters 600 HPLC system (Water scientific, USA) consisted of a Waters 717 plus auto-sampler that maintained sample temperature at 18°C, Waters 600 quaternary pump, Waters 474 fluorescence detector, and Waters 481 Lambda Max absorbance detector in series. A 2-channel data collection system (Kontron) and a flow rate of 1 mL/min were used for analysis. Calibration curves for the amino acids (0–20 nM) and AGE (0–1 nM) were constructed, and 25 μg of the control and glycated proteins were analyzed. The detection limits were 2–20 pM and the inter batch coefficients of variation ranged from 4–29% depending on the analyte. All analytical recoveries exceeded 90%.

### HPLC analysis

CML and pentosidine contents in native H1 and 3-DG-glycated H1 samples were analyzed by HPLC as previously described [43]. Briefly, the samples were first hydrolyzed with 6 N HCl for 24 h at 110°C. The hydrolyzed samples were then passed through a 0.42-μM Millex filter for ultrafiltration. The filtered samples were analyzed with an ion exchange HPLC column (2622 SC, 4.6 × 60 mm; Hitachi, Japan). The retention times of CML and pentosidine standards were measured as a reference.

### Spectroscopic analysis

UV absorbance of native and 3-DG-glycated H1 was recorded with a Perkin Elmer (Shelton, CT, USA) Lambda 25 spectrophotometer at a wavelength range of 200–400 nm [44, 33]. Percent increase of hyperchromocity was calculated with the following formula:

$$\%\;\text{increase hyperchromicity}=\frac{\text{Abs. of glycated H}1-\;\text{Abs. of native H}1}{\text{Abs. of glycated H}1}\times 100$$

### Fluorescence analysis

Fluorescence characteristics of native and 3-DG-glycated H1 were investigated with a Hitachi spectrofluorometer (F-4500). A quartz cuvette with a 1-cm pathlength was used for the experiments. Emission profiles of the samples were record at excitation wavelengths of 335 and 365 nm [44, 33]. Percent increase in the fluorescence intensity (FI) was calculated with the following formula:

$$\%\;\text{increase FI}=\frac{\text{FI of glycated H}1-\text{FI of native H}1}{\text{FI of glycated H}1}\times 100$$

### Circular dichroism (CD) measurements

A CD spectral study was performed with a Jasco spectropolarimeter, J-815 (Jasco, Japan) coupled with a Jasco Peltier-type temperature controller (PTC-424S/15). The instrument was calibrated with D-10-camphorsulphonic acid and measurements were carried out at 25°C using a temperature-controlled cell holder attached to a Neslab RTE water bath with an accuracy of ± 0.1°C. Spectral profiles of the samples (20 μM) were obtained with 1-mm pathlength cuvettes at a wavelength range of 200–250 nm. A scan speed of 100 nm/min and response time of 1 sec was selected to record the CD spectra. Two sets of each sample were analyzed under the same conditions to confirm reproducibility of the results. Furthermore, thermal unfolding of native and 3-DG-glycated H1 was evaluated by measuring the temperature-dependent CD response at 208 nm from 25–90°C at a temperature increase of 1°C/min [45, 33].

### Fourier transformed-infra red (FT-IR) spectroscopy

Structural alteration of 3-DG-glycated H1 was characterized by FT-IR spectroscopy. Transmission spectra were recorded over 600–4000 cm^-1^ with a Spectrum 100 FTIR spectrometer (Perkin Elmer) as previously described [33].

## Results

### Formation of intermediates during glycation

Free amino groups in lysine and arginine residues of native and 3-DG-glycated H1 histone were identified by fluorescamine and 9,10-phenanthrenequinone assays, respectively. The percentage of reacted lysine and arginine residues was 85.50 and 78.30% in 3-DG-glycated H1, respectively. Amadori product formation during the glycation reaction was estimated by reduction of the NBT dye. During this process, yellow NBT dye is converted to purple monoformazan, indicating the formation of Amadori products. On day 6, the maximum amount of Amadori product (12.03 ± 1.32 nM/mL) was observed in 3-DG-glycated H1. Carboxylation of lysine, arginine, threonine, and proline residues is a characteristic marker of protein oxidation. 3-DG-mediated oxidation may lead to the modification of the amino acid side chains. The carbonyl content in the 3-DG-glycated H1 sample was 23.23 ± 1.56 nmol/mg protein, while that in native H1 was 5.33 ± 0.78 nmol/mg protein. All results are summarized in Table 1.

**Table 1:** Formation of various intermediates and AGEs.

Sample	Lysine reacted (%)	Arginine reacted (%)	Carbonyl, nM/mg H1	CML, nM/ml H1 (ELISA)	CML, nM/ml H1 (HPLC)	Pentosidine nM/ml H1	Amadori product (nM/ml)
Native H1	-	-	5.33 ± 0.78	0	0	0	0
3-DG-glycated H1	85.50	78.30	23.23 ± 1.56	1.87 ± 0.20	1.73 ± 0.05	1.06 ± 0.08	12.03 ± 1.32

### Determination AGE formation by an ELISA and HPLC analysis

The CML content in native and 3-DG-glycated H1 was evaluated by ELISA immunoassay. 3-DG-glycated H1 contained 1.87 ± 0.20 nM/ml amount of CML, whereas negligible amount of CML content was observed in native H1. Significant amounts of CML and pentosidine were identified in enzymatically hydrolyzed 3-DG-glycated H1. The CML and pentosidine contents in 3-DG-glycated H1 were 1.73 ± 0.05 and 1.06 ± 0.08 nM/ml, respectively. These results are summarized in Table 1.

**Fig 1:**
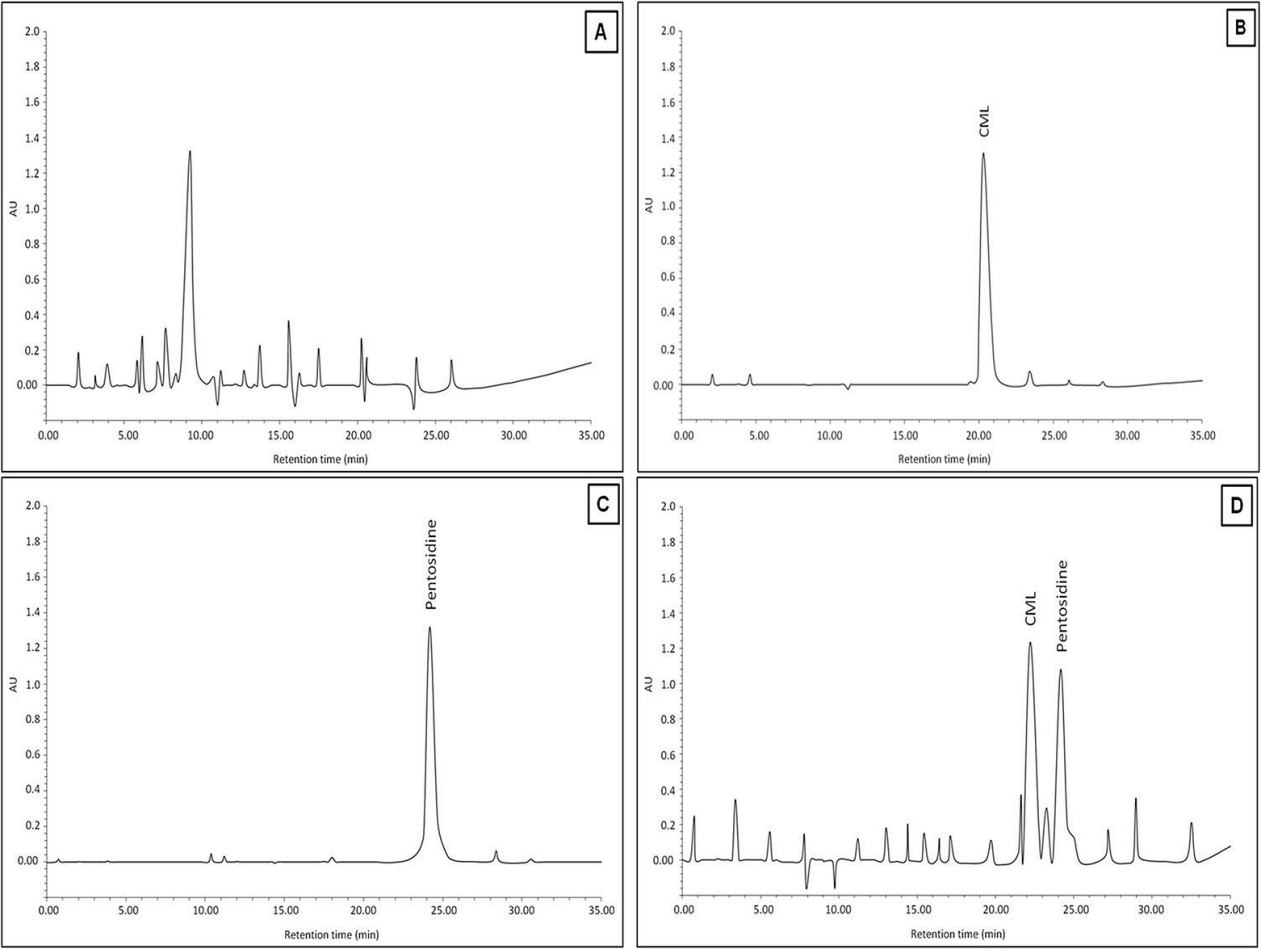
HPLC study of native and 3-DG-glycated H1 histone protein. (A) Chromatograms for native H1, (B and C) standard CML and pentosidine, and (D) glycated H1 protein.

HPLC was used to substantiate the formation of CML and pentosidine in acid hydrolyzed native and 3-DG-glycated H1. The chromatogram of native H1 did not contain any peak corresponding to standard CML or pentosidine (used as references; Fig 1A). The standard CML and pentosidine corresponded to separate peaks with retention times of 21.42 and 24.12 min, respectively (Fig 1B and 1C). Two major peaks with retention time of 23.35 and 24.18 min that correspond to standard CML and pentosidine, respectively, were observed for 3-DG-glycated H1 (Fig 1D).

### Observation of AGE formation in 3-DG-glycated H1 histone by spectroscopic analysis

**Fig 2:**
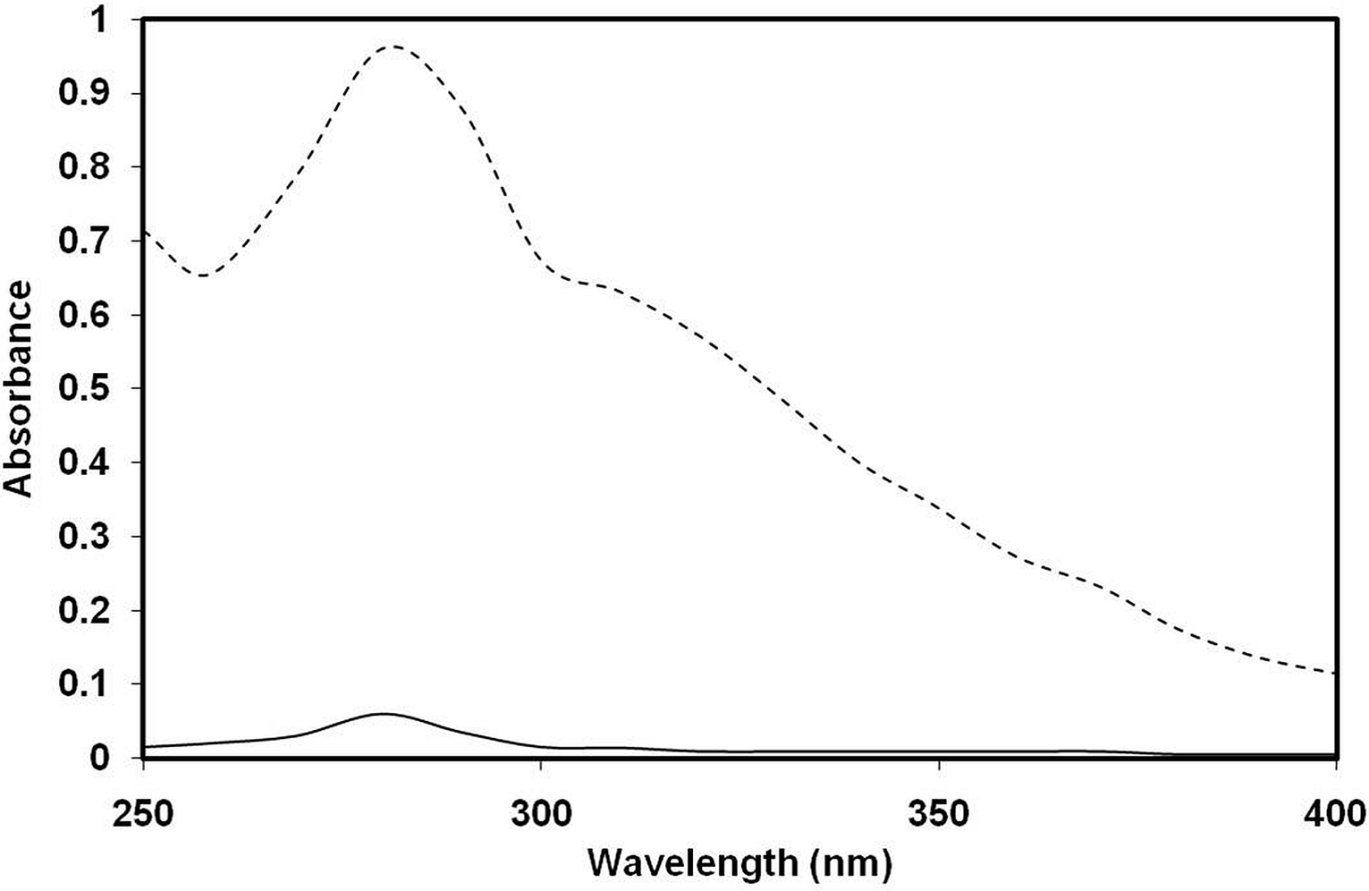
UV-Vis spectral profiles of native (—) and 3-DG- glycated H1 histone (- - - -).

**Fig 3:**
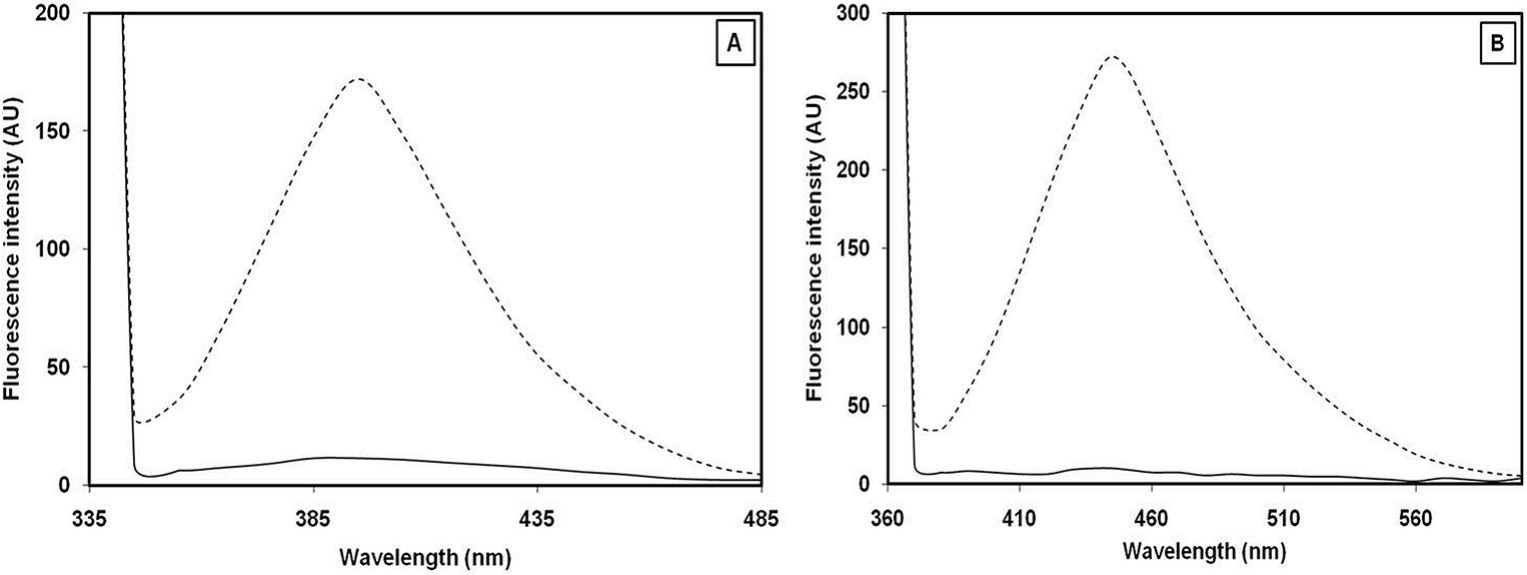
Fluorescence spectral profiles of native (—) and 3-DG-glycated H1 histone (- - -) excited at (A) 335 and (B) 365 nm.

Pilot experiments were carried out to determine the optimum concentration of 3-DG needed to modify histone protein. Briefly, 3-DG (10 μM) was mixed with H1 (1 mg/mL) and incubated at 37°C for 14 day. Native H1 produced a prominent absorbance peak at 276 nm (Fig 2). A 93.74% increase in absorbance (hyperchromicity) at 276 nm as well as increased absorbance between 300 to 400 nm was observed for 3-DG-glycated H1. Assessment of flourogenic pentosidine and formation of different AGEs in 3-DG-glycated H1 histone was investigated by exciting the samples at 335 and 365 nm [46]. 3-DG-glycated H1 had a 93.37% increase in emission intensity at λ_em_ 395 nm relative to native H1 upon excitation at λ_ex_ 335 nm (Fig 3A). The increase in fluorescence intensity at λ_em_ 395 nm for H1 glycated with 3-DG suggests the generation of pentosidine. When the same sample was again excited at 365 nm, a 96.48% increase in fluorescence intensity for glycated H1 histone was observed at λ_em_ 445 nm relative to native H1 histone (Fig 3B). Native H1 showed negligible fluorescence intensity in both cases.

### Analysis of structural alteration in 3-DG-glycated H1 protein

**Fig 4:**
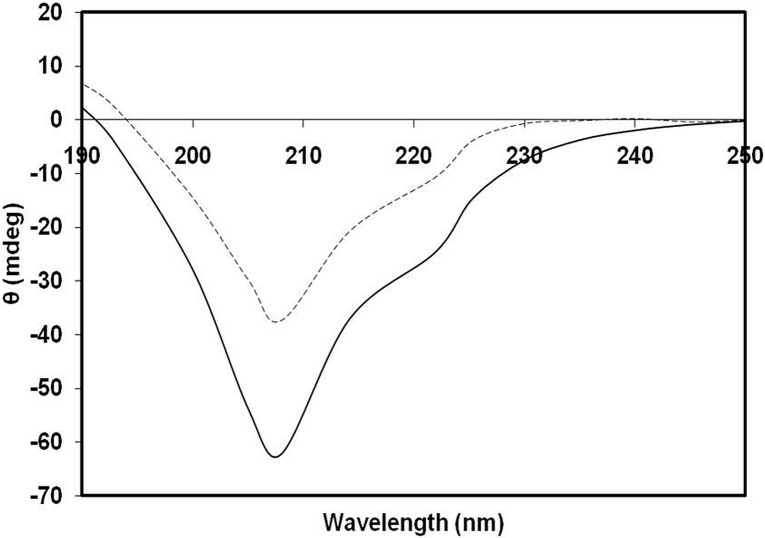
Far-UV CD spectra of native (—) and 3-DG-glycated H1 histone (- - -). The spectra were recorded between 200 and 250 nm. The protein concentration was 0.5 mg/ml and the path-length was 1.0 cm.

**Fig 5:**
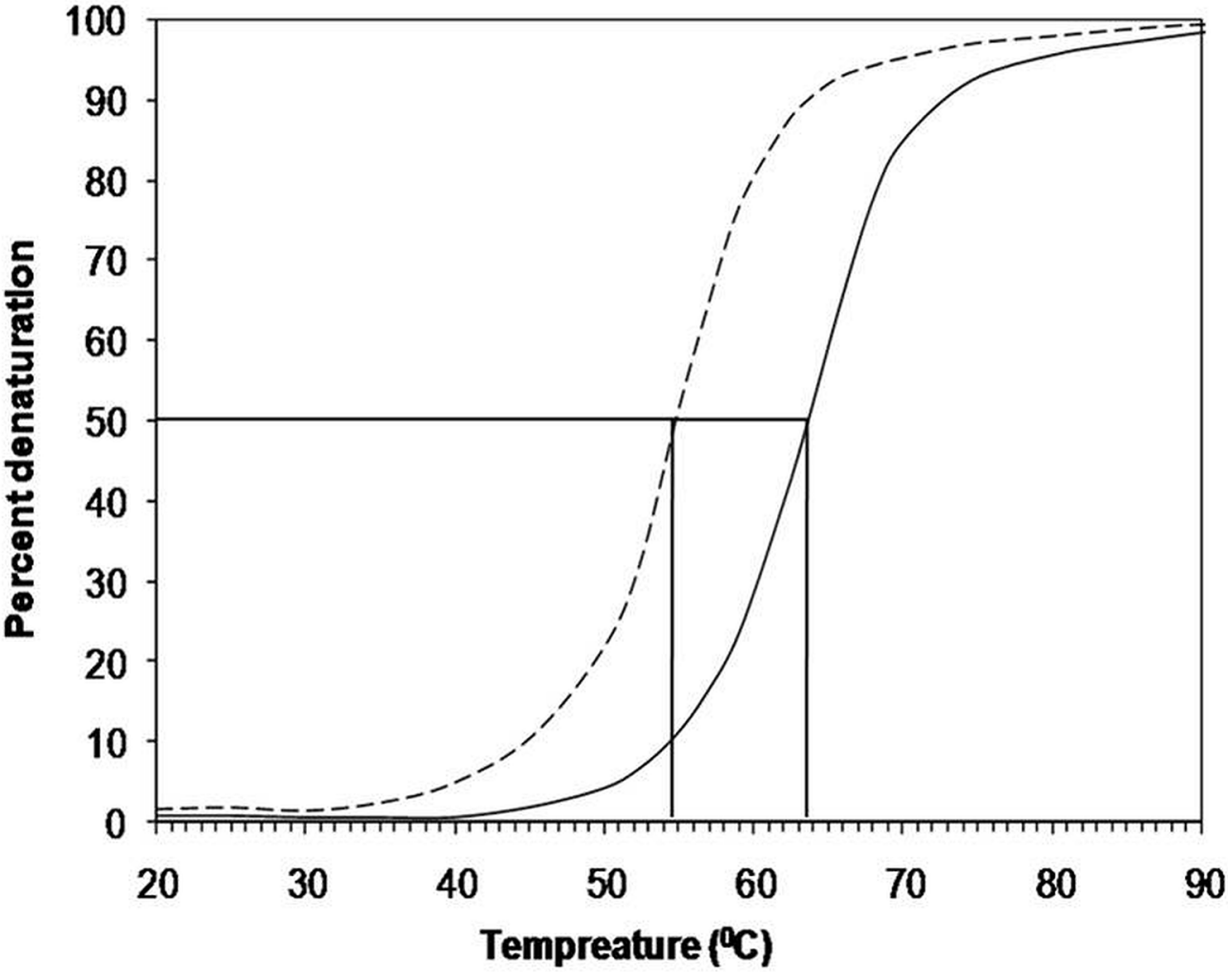
Temperature-induced denaturation spectral profiles demonstrating alteration in ellipticity at 208 nm of native (—) and 3-DG-glycated H1 histone (-—-).

CD is a powerful technique used for predicting secondary structure/conformational changes in proteins. In the present study, the characteristic CD properties of proteins were evaluated to investigate the secondary structure of native H1 and subsequent changes after modification with 3-DG. Proteins rich in α-helices produce negative bands at 222 and 208 nm. The 3-DG-glycated H1 histone had a significant decrease in ellipticity at 222 and 208 nm compared to the non-glycated counterpart (Fig 4). This decrease in ellipticity suggests that the secondary structure/conformation of 3-DG-glycated H1 was altered. Thermal unfolding was performed to check the thermodynamic stability of 3-DG-glycated H1 at 222 nm and demonstrate the presence of residual secondary structures in the protein. As shown in Fig 5, the mid-point temperature (Tm) of native H1 was 63.51°C and glycation of native H1 histone by 3-DG resulted in a significant Tm decrease (8.88°C). This significant reduction in Tm indicated a decrease in thermo stability resulting from structural changes in the 3-DG-glycated H1 protein.

**Fig 6:**
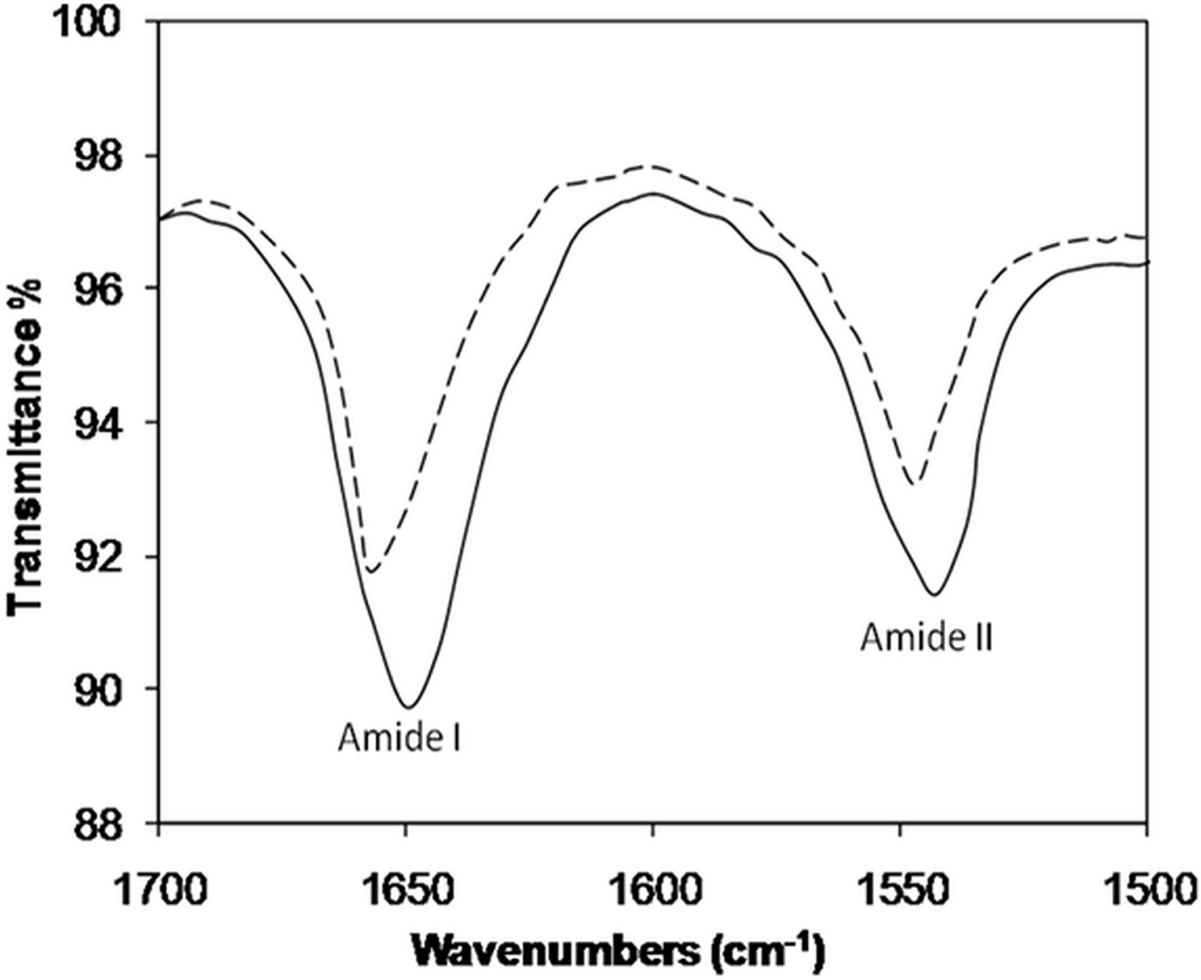
FTIR spectral profiles of Amide I and Amide II bands corresponding to native H3 histones and 3-DG-glycated H3 histone.

FT-IR is also a sensitive technique for examining secondary structural changes in proteins. The FT-IR spectra contains two transmittance bands (amide I, C = O; amide II, N-H bonds) in the range of 1500–1700 cm^-1^. The two bands are sensitive parameters that are used to predict secondary structural changes in proteins caused by chemical agents [47]. Two transmittance bands at 1651 (amide I) and 1542 cm^-1^ (amide II) were observed for native H1. After glycation, the band positions shifted to 1657 (amide I) and 1546 cm^-1^ (amide II) in the spectra (Fig 6).

## Discussion

Non-enzymatic glycation of proteins has been widely investigated and found to play a principal role in human physiology and metabolic disorders [48]. High levels of AGEs in the body are associated with a large number of diseases including secondary complications of diabetes, renal failure, and Alzheimer’s disease [49, 50]. 3-DG concentration has been reported to be higher in diabetic subjects, and AGEs consequently play a major role in the initiation and/or progression of diabetic complications [51, 52].

H1 protein is associated with DNA stretch, links nucleosome cores, and promotes the condensation of chromatin [53, 54]. A high lysine and arginine content (30%) makes it more susceptible to glycation [55]. In the present study, the ability of 3-DG to glycate H1 histone was investigated by measuring various parameters that indicate the formation of AGEs.

In our study, extent of reactivity of lysine and arginine residue indicates glycating potential of 3-DG with histone protein. Furthermore, the presence of Amadori products and carbonyl contents in 3-DG-modified H1 histone confirms the occurrence of glycation reaction. Previous study has shown that the presence of carbonyl contents point towards the oxidative modifications of proteins during the glycation reaction [56], and the presence of Amadori products signifies that the glycation reaction in its early stage [36, 40, 57]. In short, reactivity of lysine and arginine, carbonyl contents, and Amadori products detected in the reaction system are suggestive of AGE formation. Significant amounts of CML and pentosidine identified by HPLC and ELISA validate the 3-DG capacity in AGE formation. HPLC chromatogram again confirmed the formation of AGEs (CML and pentosidine), as presence of corresponding peaks to standard CML and pentosidine were observed in glycated-H1 protein.

Further fluorescence spectroscopic analysis of the samples again verified the formation of AGEs. The immense increase in fluorescence intensity observed at λ_em_ 396 nm upon excitation at λ_ex_ 335 nm suggested the formation of pentosidine during glycation [37]. Earlier study has reported that glycated arginine and lysine side chains play a key role in the formation of cross-linkage resulting in the development of pentosidine [58]. It is well documented that excitation/emission wavelengths of pentosidine are around 325–335/375–385 nm, substantiating the findings of the current study [59]. The same sample showing considerably increased fluorescence intensity emission wavelength of λ_em_ 445 nm upon excitation at λ_ex_ 365 nm indicated the formation of different AGEs with different excitation/emission wavelengths [60].

The UV absorbance profile of 3-DG-glycated H1 showed significant enhancement of absorbance (hyperchromicity) at 276 nm. This hyperchromicity could be ascribed to exposure of the chromophoric group or generation of more chromophoric groups during the glycation process. The hyperchromicity and increase in absorbance between 300 and 400 nm observed in H1 protein glycated by 3-DG are indicative of glycation taking place that leads to AGE formation [61]. These spectroscopic characteristics of macromolecules (DNA and proteins) have been attributed to glycation occurring under pathophysiological conditions like diabetes, cancer, and atherosclerosis [62–65]. Previous studies of increased absorbance in the range of 300–400 nm have been utilised to predict the formation of AGEs [16, 66].

The effect of glycation on the secondary structure of H1 histone protein was also investigated by CD and FTIR. Two minima, one at 208 nm and the other at 222 nm, an indicative of the helical contents of the protein, were observed in the analysed samples (Fig 4). A decrease in the ellipticity/upward shift in the spectrum at both minima for 3-DG-glycated H1 relative to native H1 was observed. This finding clearly indicates structural alteration or loss of α-helices upon modification by 3-DG [67]. Furthermore, a significant decrease in the Tm of modified histones at 222 nm clearly indicates a decrease in stability/loss of secondary protein structure upon glycation. Some of the interactions that stabilize compact folding conformations are disturbed in modified proteins [67]. This study agrees with previous findings from our laboratory, which demonstrates that glycation reactions and consequent AGE formation has tremendous effect on the functionality and structure of proteins [34].

Native protein structure is maintained by several types of covalent and non-covalent atomic forces. One of the basic biophysical properties of proteins is thermal denaturation vulnerability that governs protein stability and durability, and is altered upon modification [68]. Disturbance in the isoelectric point (pI) of a protein also affects the thermo-stabilizing factors due to change in charges on proteins, leading to reduced protein stability [69]. Lysine and arginine are prime targets of glycation, and are responsible for destabilization of the protein structure. FT-IR identified the structural changes in 3-DG-glycated H1, and is a sensitive technique used to investigate changes in protein structure caused by chemical modification [70]. The shift in band (amide I and amide II) positions as well as augmentation of transmission intensities compared to those of the native conformer clearly demonstrated perturbation of the secondary structure upon modification of H1 by 3-DG.

Taken together, the results of this study confirm that 3-DG is a potential glycating agent that acts through the generation of different intermediates and AGEs that consequently induce structural alteration of H1 protein. Glycation phenomenon occurring in diabetic cases may alter the structure and function of histone proteins, lead to changes in expression of genes, and compromise the function of DNA. These effects may aggravate secondary complications of diabetes. Reactive carbonyl species like glyoxal, MG, and 3-DG present a threat to the cellular system and their biological components like proteins and DNA [71]. Thus, there is a need to quench these reactive carbonyls and glycation using various medicinal plants with anti-oxidant activities [72–76], novel AGE inhibitors [16], and nano-conjugation techniques [61]. Recently, our research group paved the way to study the inhibition of glycation by silver and gold nanoparticles [61, 75]. Apart from gold and silver nanoparticles, a safe and biodegradable gelatin-based drug delivery system can also be used and has produced promising results [76].

## Supporting Information

S1 Fig Procedure of inhibition ELISA for quantification of AGEs formation. (DOC)
