# Supplementary material for: Glycation of H1 Histone by 3-Deoxyglucosone: Effects on Protein Structure and Generation of Different Advanced Glycation End Products
Source: PLoS One. 2015 Jun 29;10(6):e0130630. doi: 10.1371/journal.pone.0130630 (PMC4487796; doi:10.1371/journal.pone.0130630)
Supplement: S1 Fig — (DOC) [file pone.0130630.s001.doc]

**S1 Fig.** Inhibition ELISA procedure for quantification of AGEs formation

**
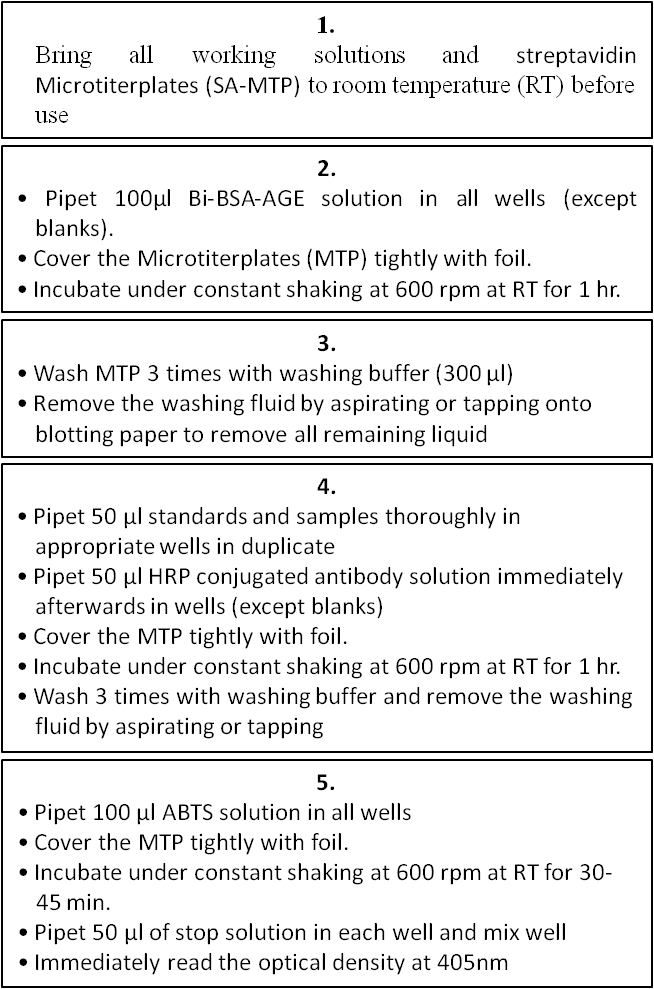
**

**Calculation of competitive ELISA graph**

1. OD and concentration of standard sample


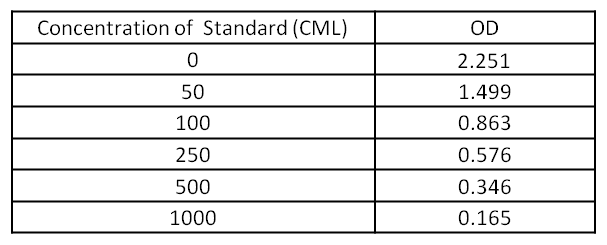


2. Samples OD


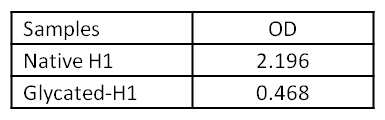


**Data Processing**

**1. Standard Curve:** Standard curve is generated by using curve software (Standard 0 is not used)


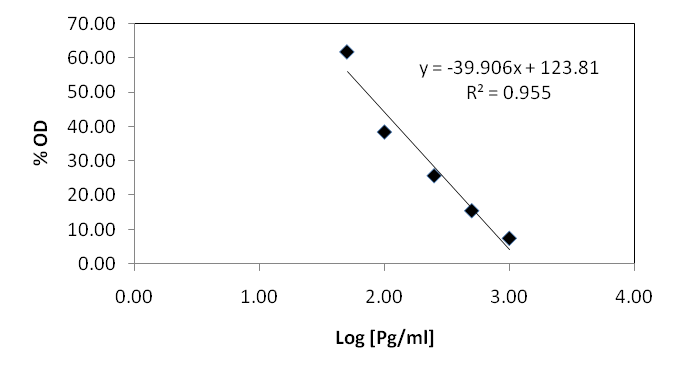


**2.** Use Linear Fit Formula to calculate the result

Linear fit formula:

Y=mx+c

**3.**  Calculation of results

[1] To process the sample OD of samples divided by that of standard 0, then multiplied by 100;

[2] To get Y value using the above formulation, Y=-39.906x+123.81

[3] To get the concentration of sample: 10 to the power of Y value


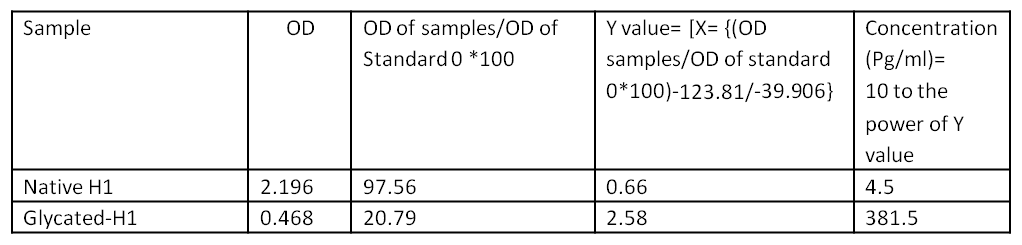


**Molar concentration of samples (Three sets):**

Native H1 (CML)= 0.02 nM ~0

Glycated-H1 (CML) = 1.87 ±0.20 nM
